# Patulin Alters Insulin Signaling and Metabolic Flexibility in HepG2 and HEK293 Cells

**DOI:** 10.3390/toxins15040244

**Published:** 2023-03-27

**Authors:** Yashodani Pillay, Savania Nagiah, Anil Chuturgoon

**Affiliations:** Discipline of Medical Biochemistry and Chemical Pathology, Faculty of Health Sciences, George Campbell Building, Howard College, University of KwaZulu-Natal, Durban 4041, South Africa

**Keywords:** insulin resistance, kidney, liver, metabolic flexibility, patulin

## Abstract

Non-communicable diseases (NCDs) have risen rapidly worldwide, sparking interest in causative agents and pathways. Patulin (PAT), a xenobiotic found in fruit products contaminated by molds, is postulated to be diabetogenic in animals, but little is known about these effects in humans. This study examined the effects of PAT on the insulin signaling pathway and the pyruvate dehydrogenase complex (PDH). HEK293 and HepG2 cells were exposed to normal (5 mM) or high (25 mM) glucose levels, insulin (1.7 nM) and PAT (0.2 μM; 2.0 μM) for 24 h. The qPCR determined gene expression of key enzymes involved in carbohydrate metabolism while Western blotting assessed the effects of PAT on the insulin signaling pathway and Pyruvate Dehydrogenase (PDH) axis. Under hyperglycemic conditions, PAT stimulated glucose production pathways, caused defects in the insulin signaling pathway and impaired PDH activity. These trends under hyperglycemic conditions remained consistent in the presence of insulin. These findings are of importance, given that PAT is ingested with fruit and fruit products. Results suggest PAT exposure may be an initiating event in insulin resistance, alluding to an etiological role in the pathogenesis of type 2 diabetes and disorders of metabolism. This highlights the importance of both diet and food quality in addressing the causes of NCDs.

## 1. Introduction

Non-communicable diseases (NCDs), including diabetes and cancer, have quickly become the leading cause of mortality worldwide, with disproportionately higher impacts on public health in developing countries [1]. This has raised interest in causative agents, diet and nutritional guidelines. Mycotoxins are secondary metabolites produced by fungi. These potentially damaging xenobiotics are found in food, with a higher incidence in developing countries [2,3]. 

Patulin (PAT) is a mycotoxin produced by *Penicillium, Bissochlamys* and *Aspergillus* sp. [4]. These molds are found in overripe or rotting fruit and apple products. A safety level of 50 μg/L PAT in consumables was established following evidence of adverse effects in exposed humans and animals [3,5]. Despite this regulation and the assertion that PAT levels are a measure of poor product quality, there are vast variations in PAT concentrations in apple products worldwide [5]. PAT induces oxidative stress, compromises mitochondrial function and causes cell death via depletion of antioxidant glutathione (GSH) [6,7,8,9,10,11]. PAT is also associated with changes in lipid metabolism and inflammation [2,12]. These deleterious effects are most commonly observed in organs of high metabolic demand—the kidney, gastrointestinal tract, liver and brain [3,13].

Energy demand and glucose homeostasis are typically maintained through the catabolism of glucose to pyruvate during glycolysis. Pyruvate is subsequently taken up by the mitochondria and broken down in the citric acid (TCA) cycle; subsequently, this facilitates the transfer of electrons to the mitochondrial respiratory chain for ATP production via oxidative phosphorylation. Under hypoxic and hypoglycemic conditions, however, these processes are impaired. These conditions divert metabolic operations to produce glucose through gluconeogenesis and glycogenolysis, resulting in ATP production via anaerobic glycolysis [14]. This metabolic environment is potentially simulated by PAT exposure which has been linked to glycogen breakdown, gluconeogenesis, impaired mitochondrial function and ATP levels in previous studies [15].

Metabolic flexibility is determined by an organism’s ability to adapt fuel oxidation processes to nutrient availability and rapidly switch between them. Pyruvate Dehydrogenase (PDH), a multienzyme complex stimulated by insulin catalyzes the conversion of pyruvate to acetyl-CoA, connecting the TCA cycle to glucose and fatty acid metabolism. PDH activity is thus a central component of metabolic flexibility. The pyruvate dehydrogenase kinases (PDKs) inhibit PDH activity through phosphorylation on the PDH E1α subunit under hypoglycemic and hypoxic conditions. PDK elevation is hence associated with gluconeogenesis, metabolic inflexibility and has been implicated in the etiology of type 2 diabetes [16,17].

Glucose uptake in mammalian cells is facilitated by a family of glucose transporters (GLUTs). GLUT2, expressed in the intestine, liver, kidney and pancreatic beta cells, is responsible for glucose sensing and uptake. Loss of GLUT2 expression leads to organ damage, persistent hyperglycemia and diabetes [18]. Insulin promotes the uptake, utilization and storage of glucose by stimulating glycolytic enzyme phosphofructokinase (PFK) and repressing gluconeogenesis and glycogenolysis enzymes, phosphoenolpyruvate carboxykinase (PCK) and glycogen phosphorylase (PYGL) [19]. Insulin activity is initiated by the binding and autophosphorylation of tyrosine residues on the insulin receptor (IR). The activated receptor phosphorylates the insulin receptor substrate 1 (IRS-1). Phosphorylated IRS-1 (pIRS-1) recruits phosphatidylinositide (PI) 3-kinase (PI3K) (p85/p110 subunits) to the plasma membrane which stimulates PI3K-mediated phosphorylation of Akt. Glycogen synthesis kinase (GSK-3) is phosphorylated and inactivated by pAkt; relieving its inhibitory phosphorylation of glycogen synthase (GS), promoting glycogen synthesis. pIRS-1 activates the extracellular signal-regulated kinases (ERK) and mitogen-activated protein kinases (MAPK) pathway in parallel which can be used as a marker for the activation of proximal insulin signaling [20,21]. Underlying insulin resistance in type 2 diabetes has been attributed to defects in one or more components of the insulin signaling cascade [22]. Thus, while previous studies have shown PAT causes changes in PI3K/Akt and ERK/MAPK signaling, little is understood about the metabolic implications thereof [23]. 

Elevated glucose levels resulting from hepatic glucose production are a feature of type 2 diabetes. Insulin resistance precedes increased PCK and PYGL expression causing glucose efflux and hyperglycemia [19]. Previous studies in animals have referred to PAT as diabetogenic describing elevated glucose levels, depleted glycogen stores and mitochondrial dysfunction following PAT exposure, but mechanistic data in human models are lacking [15,24,25]. PAT toxicity has been described previously in the liver and kidney—both of which have high metabolic demands and are regulators in glucose homeostasis [11,17,26,27]. This study aimed to determine the effects of PAT on insulin signaling and metabolic flexibility in HepG2 and HEK293 cells. 

## 2. Results

### 2.1. PAT Alters Expression of Enzymes Involved in Glucose Generation

Previous studies have alluded to PAT as a diabetogenic agent [15]. In a preliminary screening, HEK293 and HepG2 cells were exposed to a high glucose (25 mM) medium and PAT for 24 h. qPCR was then used to measure gene expression of enzymes vital to glucose homeostasis. 

**Figure 1:**
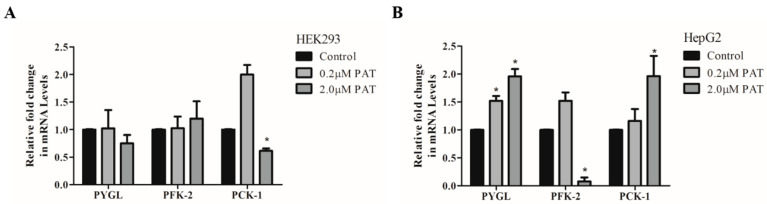
PAT alters expression of enzymes involved in glucose production in hyperglycemic conditions. qPCR was used to assess expression of glucose homeostasis enzymes *PYGL, PFK-1* and *PCK-1* in (**A**) HEK239 and (**B**) HepG2 cells exposed to 25 mM glucose and PAT for 24 h (* *p* < 0.05 relative to control).

*PFK-2*, a key enzyme in glycolysis, was not altered by PAT in HEK293 cells (Figure 1A; *p =* 0.7270) but decreased significantly in HepG2 cells (Figure 1B; *p =* 0.0231; 10-fold). Although overall statistical significance was found for glycogenolysis initiator, *PYGL*, in both cell lines (HEK293 (Figure 1A; *p =* 0.0137)), post-hoc tests found that only the distinct two-fold increase in HepG2 cells was significant when compared to the control (Figure 1B; *p =* 0.0218)]. *PCK-1*, a rate limiting enzyme in gluconeogenesis, was significantly altered by PAT at 2 μM in both cell lines, though trends differed between them (HEK293 cells (Figure 1A; *p =* 0.0112) and HepG2 cells (Figure 1B; *p =* 0.0214)). This outcome provides a preliminary indication that PAT caused a shift from glycolysis to gluconeogenesis and glycogenolysis in HepG2 cells with HG media. Most significant changes were observed at 2 μM PAT–above the established safety concentration for PAT in consumables.

### 2.2. Glucose Availability Determines PAT-Associated Insulin Signaling Defects in HepG2 Cells

**Figure 2:**
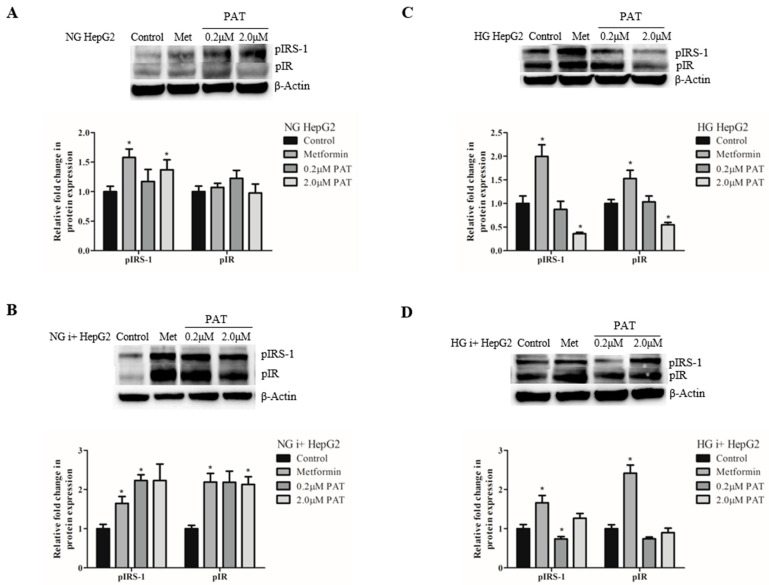
PAT alters insulin signaling in HepG2 cells. Western blotting determined PAT-increased pIR and pIRS-1 in NG media (**A**) and under insulin stimulation (NG i+) (**B**). This effect was reversed in HG media (**C**) decreased pIRS-1 and pIR expression was observed which remained consistent under insulin-stimulated hyperglycemic conditions (HG i+) (**D**) (* *p* < 0.05 relative to control).

IR activation triggers phosphorylation of the receptor and IRS-1. Under NG conditions, PAT stimulated IRS-1 phosphorylation (pIRS-1) (Figure 2A; *p* = 0.0388), though pIR showed no statistical change (Figure 2A; *p* = 0.0775). This trend was potentiated in the presence of insulin shown by a significant two-fold increase in pIR (Figure 2B; *p* = 0.0180) and pIRS-1 (Figure 2B; *p* = 0.0145) in PAT-exposed NG i+ HepG2 cells, exhibiting a similar effect to the positive control, metformin. 

This observation was dramatically reversed under hyperglycemic conditions. PAT caused significant two-fold decreases in both pIR (Figure 2C; *p =* 0.0178) and pIRS-1 (Figure 2C; *p =* 0.0213) in HG HepG2 cells. This result was consistent following insulin pathway stimulation (HG i+ HepG2) where both pIR (Figure 2D; *p =* 0.0182) and pIRS-1 (Figure 2D; *p =* 0.0188) were decreased in PAT treatments. These findings are suggestive of defects in insulin signaling under HG conditions following PAT exposure. 

### 2.3. PAT-Altered ERK/MAPK Signaling Is Influenced by Glucose and Insulin Availability

**Figure 3:**
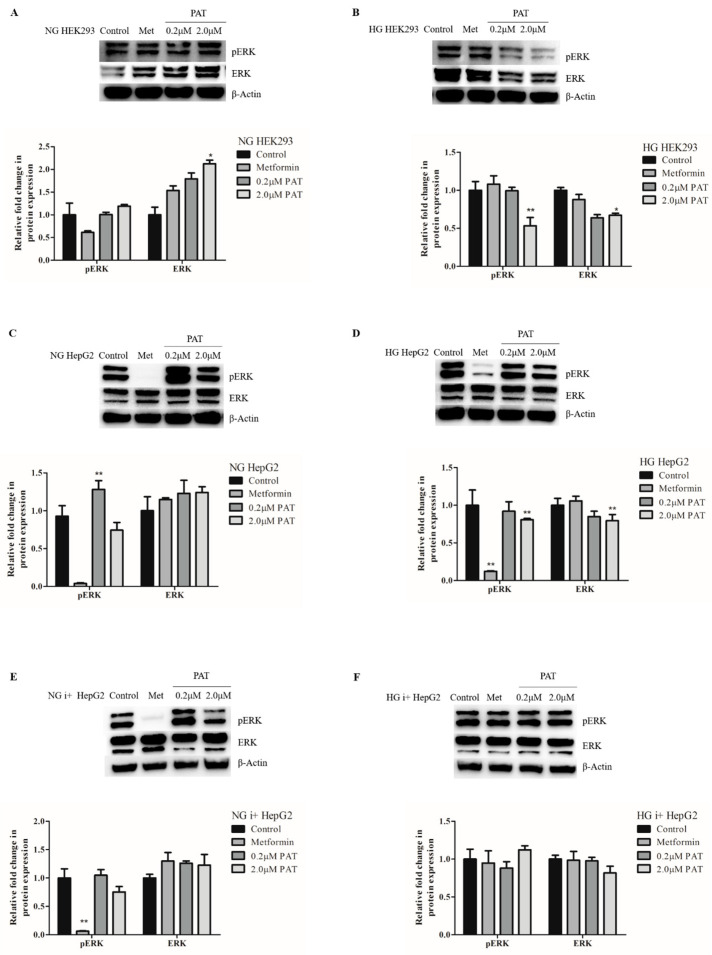
PAT alters ERK/MAPK according to glucose and insulin availability. Western blotting determined PAT caused a significant increase in ERK1/2 phosphorylation activation relative to total ERK expression in NG HEK293 (**A**) and NG HepG2 (**C**) cells. This was reversed in HG HEK293 (**B**) and HG HepG2 (**D**) cells with decreases in pERK and ERK following 24 h PAT exposure. Both trends were neutralized in the presence of insulin (**E**,**F**) (** p < 0.01, * *p* < 0.05 relative to control).

Insulin-activated ERK phosphorylation is connected to the downstream regulation of transcription factors involved in metabolic homeostasis. Under NG conditions, PAT increased ERK activation in both HEK293 and HepG2 cells evidenced by increased pERK relative to total ERK (NG HEK293 (Figure 3A; pERK: *p =* 0.00610; ERK; *p =* 0.0032); NG HepG2 (Figure 3C; pERK: *p =* 0.0072; ERK: *p =* 0.0788)).

Conversely, under HG conditions, PAT-exposed HEK293 cells significantly decreased both pERK (Figure 3B; *p =* 0.0221) and total ERK (Figure 3B; *p =* 0.0053) expression. This observation was consistent in HG HepG2 cells (Figure 3D; pERK: *p =* 0.0104 and ERK: *p =* 0.0149). Interestingly, the effects of PAT on ERK signaling in HepG2s under both NG and HG were neutralized in the presence of insulin. No significant effects on ERK activation were observed in PAT-exposed NG i+ HepG2 (Figure 3E; pERK: *p =* 0.0630 and ERK: *p =* 0.0399) or HG i+ HepG2 (Figure 3F; pERK: *p =* 0.2027; and ERK: *p =* 0.0920) treatments. 

PAT-altered ERK signaling trends were dissimilar to positive control metformin results, indicating a potential alternate mechanism of action. These data suggest that PAT stimulates ERK/MAPK signaling under NG conditions and inhibits signaling under HG conditions. These effects can, however, be counteracted by stimulating the insulin signaling pathway (Figure 3).

### 2.4. Glucose Availability Affects PAT-Mediated PI3K/Akt Signaling Trends

**Figure 4:**
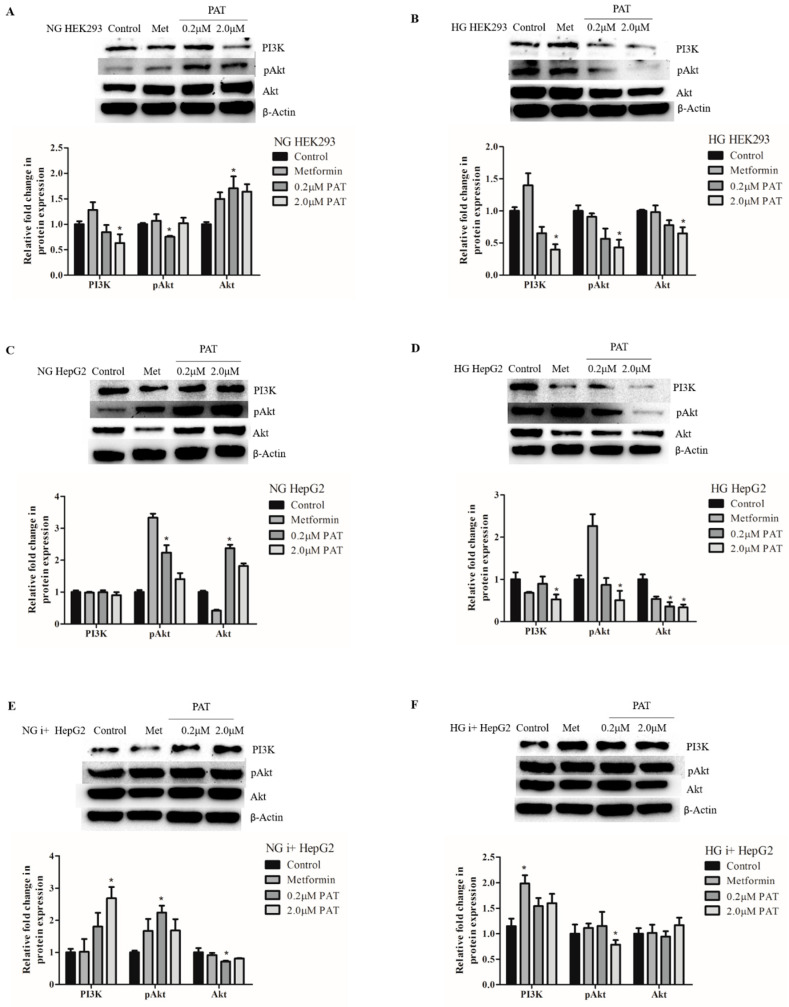
PAT alters PI3K/Akt signaling according to glucose availability irrespective of insulin action. Western blotting established PAT significantly increased PI3K activation and Akt phosphorylation activation relative to total Akt expression in NG HEK293 (**A**) and NG HepG2 (**C**) cells. This was reversed in HG HEK293 (**B**) and HG HepG2 (**D**) cells with decreases in PI3K, pAkt following 24 h PAT exposure. Both trends were maintained in the presence of insulin (**E**,**F**) (* *p* < 0.05 relative to control).

Insulin-stimulated PI3K activity directs the phosphorylation and activation of downstream target Akt. In PAT-treated NG HEK293 cells, PI3K (Figure 4A; *p* = 0.0051) and pAkt (Figure 4A; *p* = 0.0329) were significantly decreased while total inactive Akt (Figure 4A; *p =* 0.0160) increased. However, in NG HepG2 and NG i+ HepG2 cells PAT significantly increased the PI3K/Akt pathway activation evidenced by increased PI3K and pAkt expression relative to total Akt (NG HepG2 (Figure 4C; PI3K: *p =* 0.1431; pAkt: *p =* 0.0043; Akt: *p =* 0.0027); NG i+ HepG2 (Figure 4E; PI3K: *p =* 0.0112; pAkt: *p =* 0.0249; Akt: *p =* 0.0237)).

Conversely, PI3K/Akt pathway activation significantly decreased under all PAT HG conditions (HG HEK293 (Figure 4B; PI3K, *p =* 0.0043; pAkt, *p =* 0.0072; Akt, *p =* 0.0092); HG HepG2 (Figure 4D; PI3K, *p =* 0.0219; pAkt, *p =* 0.0115; Akt, *p =* 0.0053); HG i+ HepG2 (Figure 4F; PI3K: *p =* 0.0160; pAkt: *p =* 0.0415; Akt: *p =* 0.1681)). 

These results show PAT stimulated PI3K/Akt signaling under NG conditions and inhibited PI3K/Akt activation under HG conditions despite insulin action. This indicated a possible PAT-induced defect in response to elevated glucose levels and insulin-stimulated signaling. 

### 2.5. PAT-Modified GSK-3 Activation Corresponded with PI3K/Akt Signaling Trends While GLUT2 Expression Was Widely Compromised by PAT

**Figure 5:**
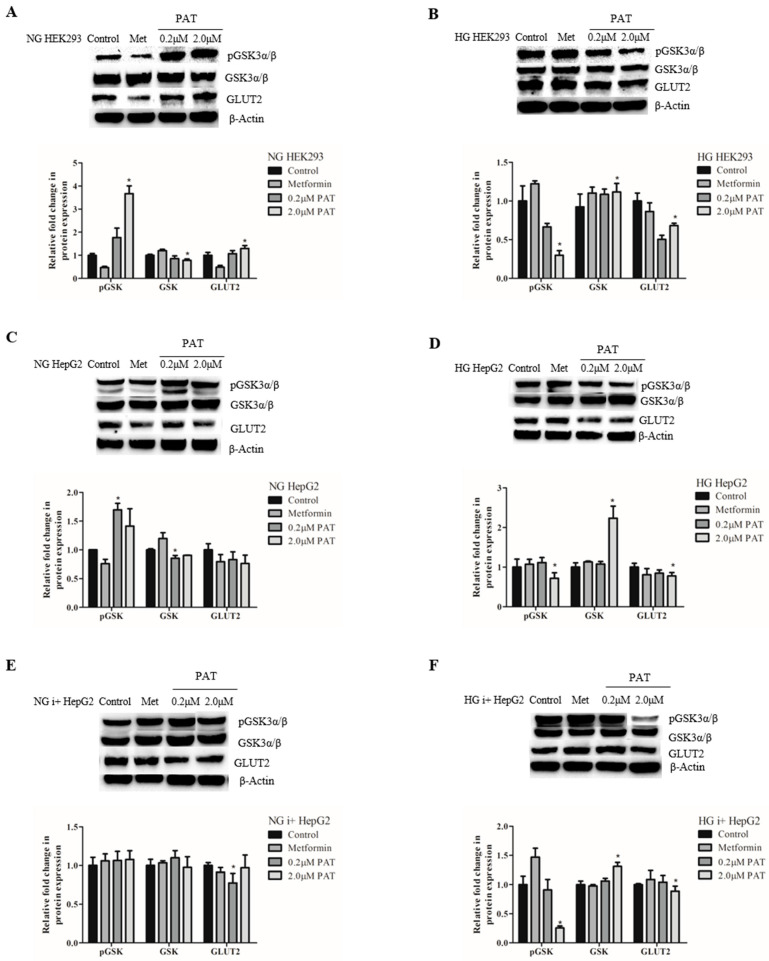
PAT-modified GSK-3 activation corresponded with PI3K/Akt signaling trends while GLUT2 expression was widely compromised by PAT. Western blotting established PAT significantly increased GSK-3 inhibition by phosphorylation relative to total GSK-3 expression with no change GLUT2 in NG HEK293 (**A**) and NG HepG2 (**C**) cells. This was reversed in HG HEK293 (**B**) and HG HepG2 (**D**) cells with decreases in GLUT2, pGSK-3, and increased GSK-3 following 24 h PAT exposure. NG i+ cells (**E**) exposed to PAT showed no significant changes while HG trends were maintained in the presence of insulin (**F**) (* *p* < 0.05 relative to control).

Active GSK-3 phosphorylates and inhibits GS, preventing glycogen synthesis. Insulin action phosphorylates and inhibits GSK-3 (pGSK-3) via PI3K/Akt signaling, which enhances glycogen synthesis. Under NG conditions, PAT significantly increased pGSK-3 and decreased total active GSK-3 in both HEK293 and HepG2 cells suggestive of decreased GSK activity (NG HEK293 (Figure 5A; pGSK-3: *p =* 0.0073; GSK-3: *p =* 0.0488); NG HepG2 (Figure 5C; pGSK-3: *p =* 0.0415; GSK-3: *p =* 0.0216)). 

In HG, the media effects were reversed, exhibiting GSK-3 activation in both cell lines. In HG HEK293 cells, inactive pGSK-3 decreased (Figure 5B; *p =* 0.0158) and total active GSK-3 increased (Figure 5C; *p =* 0.0216). This effect was more pronounced in PAT-treated HG HepG2 cells (Figure 5D; pGSK-3: *p =* 0.0158; GSK-3: *p =* 0.0149)—indicative of compromised glycogen synthesis. When stimulated with insulin, inactive pGSK-3 remained decreased in HG i+ HepG2 cells (Figure 5F; *p =* 0.0056). No changes were noted in NG i+ HepG2 cells in either pGSK-3 (Figure 5E; *p =* 0.3815) or GSK-3 (Figure 5E; *p =* 0.4969). These results suggest PAT caused defects in the GSK-3 signaling axis under HG conditions.

GLUT2, involved in glucose sensing and uptake, is linked to impaired glucose-stimulated insulin signaling when suppressed. PAT did not alter GLUT2 expression under NG conditions in both HEK293 and HepG2 cells (NG HEK293 (Figure 5A; *p =* 0.0556); NG HepG2 (Figure 5C; *p =* 0.0559)). In the presence of glucose and insulin, however, PAT significantly decreased GLUT2 expression in both cell lines (NG i+ HepG2 cells (Figure 5E; *p =* 0.0496); HG HEK293 (Figure 5B; *p =* 0.0048); HG HepG2; (Figure 5D; *p =* 0.0077); HG i+ HepG2 cells (Figure 5F; *p =* 0.0281)). This suggests PAT impaired GLUT2-mediated glucose sensing and uptake under conditions of increased glucose and insulin availability.

### 2.6. PAT Contributes to Metabolic Inflexibility by PDK-1 Elevation and PDH Inhibition under NG and HG Conditions

Active PDH, stimulated by insulin, catalyzes the conversion of pyruvate to acetyl-CoA. Inhibition of PDH is catalyzed by PDK-mediated phosphorylation on the E1α subunit. 

**Figure 6:**
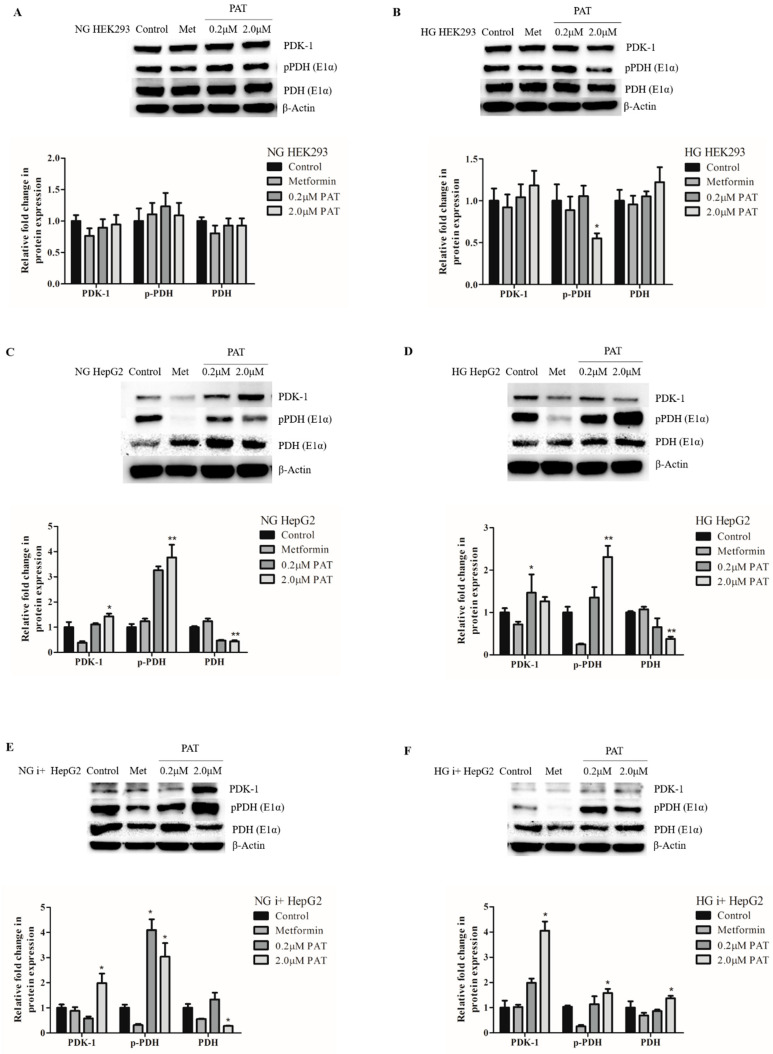
PAT contributes to metabolic inflexibility by PDK-1 elevation and PDH inhibition under NG and HG conditions. Western blotting established PAT caused no significant differences in PDK-1, pPDH E1α and PDH E1α in NG HEK293 (**A**) and HG HEK293 (**B**) cells. PDK-1 was significantly increased with associated changed in pPDH E1α and PDH E1α in NG HepG2 (**C**), HG HepG2 (**D**) NG i+ HepG2 (**E**) and HG i+ HepG2 (**F**) cells following 24 h PAT exposure. (** *p* < 0.01, * *p* < 0.05 relative to control).

PAT did not alter PDK-1 and PDH E1α expression in HEK293 cells (NG (Figure 6A; PDK-1: *p =* 0.1390; PDH E1α: *p =* 0.2220); HG (Figure 6B; PDK-1: *p =* 0.2223; PDH E1α: *p =* 0.1417)). pPDH E1α expression remained unchanged in NG HEK293 cells (Figure 6A; *p =* 0.3588) but decreased in HG HEK293 cells (Figure 6B; *p =* 0.0221). 

PAT-mediated effects on this pathway were more distinct in HepG2 cells. PDK-1 expression significantly increased in PAT-treated HepG2 cells under all conditions (NG (Figure 6C; *p =* 0.0060), HG (Figure 6D; *p =* 0.0186), NG i+ (Figure 6E; *p =* 0.0048), HG i+ (Figure 6F; *p =* 0.0081)). An associated increase in (inactive) pPDH E1α (NG (Figure 6C; *p =* 0.0045), HG (Figure 6D; *p =* 0.0041), NG i+ (Figure 6E; *p =* 0.0041) and HG i+ (Figure 6F; *p =* 0.0179)) with a concomitant decrease in PDH E1α (NG (Figure 6C; *p =* 0.0060), HG (Figure 6D; *p =* 0.0055), NG i+ (Figure 6E; *p =* 0.0032) and HG i+ (Figure 6F; *p =* 0.0124)) was observed under these conditions in PAT treatments. This was indicative of PDK-1-mediated PDH inhibition in PAT-treated HepG2 cells.

Elevated pPDH E1α suppresses aerobic pyruvate oxidation and can aggravate the Warburg effect. This pathway, characterized predominantly in cancerous cells, favors the anaerobic conversion of pyruvate to lactate. This may explain the distinct differences in PDH E1α results between HepG2 (cancerous) and HEK293 (non-cancerous) cells. Interestingly, lactate levels increased significantly in controls under all HG conditions relative to NG (Table 1; (HEK293; *p =* 0.0006) (HepG2; *p =* 0.0013) (HepG2 i+; *p =* 0.0190)). The highly significant increase observed in HEK293 cells relative to HepG2 cells may be related to metabolic differences in cancerous and non-cancerous cells as well as the pH sensitivity of HEK293 cells. Interestingly, a significant decrease in lactate levels was observed in PAT treatments relative to the controls in NG treatments and HEK293 cells (Table 1; NG HEK293 (*p =* 0.0222); HG HEK293 (*p =* 0.0069); NG HepG2 (*p =* 0.0142); NG i+ (*p =* 0.0069)). HG HepG2 and HG i+ HepG2 treatments however, (Table 1; NG HepG2 (*p =* 0.4336); NG i+ (*p =* 0.7427)) were not statistically changed. This lack of lactate elevation despite changes to the PDH axis in PAT treatments suggests pyruvate was possibly shunted back towards gluconeogenesis or to an alternative fate instead of reduction to lactate by LDH. 

## 3. Discussion

The key finding in this study is that exposure to PAT impairs insulin signaling under HG conditions and alters metabolic flexibility after 24 h exposure in an in vitro model. This finding was consistent following insulin stimulation, suggestive of an insulin-resistant mechanism.

A recent study showed PAT was cytotoxic to rat pancreatic β cells but did not affect insulin production [24,25] prompting examination of the insulin signaling cascade in this study. The IR, a tyrosine kinase, undergoes autophosphorylation catalyzing the phosphorylation and activation of downstream cellular proteins including IRS-1, the PI3K/Akt pathway and ERK/MAPK pathway. These pathways act in concert to positively regulate glycolysis, glucose storage as glycogen and lipids and repress glucose synthesis and release by inhibiting glycogenolysis and gluconeogenesis. Insulin action is attenuated by dephosphorylation of the receptor and its substrates. Phospho-tyrosine residues indicated PAT activated the insulin signaling cascade under NG conditions but suppressed activation under HG conditions in HepG2 cells (Figure 2). Defects in this pathway have been linked to impaired glucose tolerance and the pathogenesis of type 2 diabetes [22,28].

The ERK/MAPK pathway is activated in a stepwise sequence within the insulin signaling cascade. pERK catalyzes the activation of transcription factors to initiate cell proliferation. Inhibition of this pathway observed in PAT exposed HG HepG2 and HEK293 cells (Figure 3) prevents insulin-stimulated cell growth. PAT-induced alterations in ERK signaling and proliferation have been reported in the literature, but metabolic consequences have been neglected [4,23]. The findings in this study imply PAT-mediated ERK changes are dependent on insulin and glucose availability (Figure 3). This may be of consequence to glucose metabolism under conditions of PAT-inflicted β-cell death [24].

Proximal insulin signaling activates the PI3K/Akt pathway in parallel to the ERK/MAPK pathway. pIRS-1 recruits PI3K to activate Akt by phosphorylation. pAkt-mediated phosphorylation of GSK-3 (ser 9) relieves GS inhibition and promotes glycogen synthesis. Under NG conditions, PAT-activated pIRS-1 (Figure 2), PI3K/Akt activation (Figure 4) and increased pGSK-3 (Figure 5) in HepG2 cells positively regulating glycogen synthesis. Under HG conditions, however, PAT diminished pIRS-1 (Figure 2) in HepG2 cells and PI3K/Akt (Figure 4) mediated GSK-3 (Figure 5) phosphorylation in both HepG2 and HEK293 cells. These findings reveal distinct differences in PAT-mediated signaling depending on glucose availability. PAT-altered PI3K/Akt signaling has been reported previously in other cell lines [23]; the findings in this study, however, reveal novel metabolic implications. These trends identified in HepG2 cells were observed consistently in both the presence and absence of insulin, implicating a non-insulin dependent mechanism. Elevated total GSK-3 is associated with impaired glucose disposal and glycogen synthesis and type 2 diabetes [20]. Similar dysfunctions in reduced pIRS-1/PI3K/Akt signaling as observed in this study, have been associated with insulin resistance and impaired glucose tolerance [21,29]. 

Previous studies describe PAT-inflicted glycogen loss and serum glucose elevation in animals, postulating that PAT increases gluconeogenesis and glycogenolysis [15,30]. In this study, active GSK-3, a prominent feature in glycogenolysis, is elevated by PAT under HG conditions in both HEK293 and HepG2 cell lines (Figure 5). PAT also increased the gene expression of glucose-producing enzymes *PYGL* and *PCK-1* and inhibited glycolytic *PFK-1* (Figure 1) under the same conditions in HepG2s. These classic indicators of glycogenolysis and gluconeogenesis, usually inhibited by insulin and HG conditions [28], were increased by PAT under the same conditions. Glucose homeostasis relies on the detection of varying glucose availability. GLUT2, expressed in the kidney and liver, plays a crucial role in glucose sensing and uptake. GLUT2 suppression, induced by PAT in both HEK293 and HepG2 cells (Figure 5), is associated with cellular glucose efflux, impaired activity of glucose sensitive genes, reduced glucose uptake and promoting type 2 diabetes pathogenesis and organ damage [18,31]. While organ damage has been described in previous findings on PAT, this novel finding offers a possible explanation for the increased gluconeogenic markers, impaired glycogen synthesis and glycolysis in PAT treatments in this study. 

These findings indicate that PAT compromised cellular metabolic processes in adaption to glucose availability. PDH, the principal enzyme controlling nutrient adaption and metabolic flexibility, is activated by insulin and glucose via dephosphorylation on the E1α subunit. PDH metabolizes pyruvate at the junction between glucose, fatty acid metabolism and the TCA cycle [17]. PDK-1 phosphorylates and inhibits PDH E1α under hypoxic conditions, shifting metabolism to glycolytic processes [32]. PAT significantly increased PDK-1 and pPDH E1α expression in HepG2 cells (Figure 6). In cancerous cells such as HepG2s, this is associated with aggravation of the Warburg effect. The Warburg effect posits cancerous cells inhibit mitochondrial ATP production and increase rates of glycolysis and cytoplasmic conversion of pyruvate to lactate [33,34]. This pathway is linked to glucose scarcity, synthesis and inhibition of pyruvate conversion to acetyl-CoA. Mitochondrial inhibition in cancerous cells is achieved by PDK and pPDH elevation. These actions block excessive mitochondrial ROS production, linked extensively to PAT in previous studies [14]. Thus, PAT-induced pPDH and PDK-1 elevation may be an energy- and mitochondrial conservation strategy [4,10,11]. In addition, lactate levels were decreased in PAT treatments (Table 1), despite PDK-1 and pPDH E1α (Figure 6) elevation. While the lower levels of lactate in i+ treatments suggest insulin may have stimulated mitochondrial function, the high levels in the HEK293 control cells may explain the potent toxicity of PAT in the cell line as PAT loses biological activity in alkaline media [35,36]. Taken together, the results imply pyruvate did not undergo lactic acid fermentation as suggested in the Warburg effect theory. Alternate fates of pyruvate under PDH inhibition include the TCA cycle, fatty acid synthesis and gluconeogenesis [17]. While there is currently insufficient evidence in this study and the literature to conclusively rule out the TCA cycle or fatty acid synthesis these findings, together with gluconeogenic enzyme mRNA levels (Figure 1), GLUT2 and GSK-3 expression (Figure 5), infer pyruvate was shunted back toward gluconeogenesis despite the availability of glucose and insulin. Decreased PDH and metabolic inflexibility are initiating events in impaired glucose oxidation, associated with insulin resistance and type 2 diabetes [14,16,37,38,39].

Type 2 diabetes is characterized by elevated glucose levels, impaired glucose tolerance, insulin resistance, defects in the insulin signaling pathway, oxidative stress and mitochondrial dysfunction [22]. The literature shows PAT causes oxidative stress and mitochondrial dysfunction which is associated with both causes and consequences of insulin resistance [10,11,26,40]. This study presents novel findings indicating PAT opposes insulin action under HG conditions, suppresses components of the insulin signaling pathway and prevents metabolic flexibility in HepG2 and HEK293 cells. Given that PAT targets the kidney and liver, and the role of these organs in systemic glucose and energy homeostasis, this has strong implications for PAT-induced toxicology and disorders of metabolism.

## 4. Conclusions

Our findings show PAT caused defects in the insulin signaling pathway under HG conditions, stimulated glucose production pathways and inhibited PDH activity, contributing to metabolic inflexibility. Collectively, these results suggest PAT exposure may be an initiating event in insulin resistance with an etiological role in pathogenesis of type 2 diabetes and other disorders of metabolism. Significant results were observed above and below the safety level of PAT.

## 5. Materials and Methods

HEK293 and HepG2 cells were obtained from the American Type Culture Collection (Johannesburg, South Africa). Culture reagents were purchased from Lonza BioWhittaker (Basel, Switzerland). Western Blotting and qPCR reagents and consumables were purchased from Bio-Rad (Hercules, CA, USA). Patulin (P1639) was purchased from Sigma-Aldrich (St. Louis, MO, USA). All other reagents were obtained from Merck (Darmstadt, Germany) unless otherwise stated. 

### 5.1. Cell Culture

HepG2 cells were cultured in complete culture medium (CCM) containing Eagle’s minimum essential medium (EMEM) supplemented with 2 mM l-glutamine, 1% pen-strep-fungizone (500 units potassium penicillin, 500 μg streptomycin, 1.25 μg amphotericin B/5 mL flask) and 10% fetal bovine serum in 25 cm^3^ flasks at 37 °C. HepG2 cells were derived from hepatocellular carcinoma. Despite the limitations of being a cancerous line (relative to primary human hepatocytes), HepG2 cells were used as they exhibit phenotypic stability, genotypic features of normal liver cells and exhibit high glucose consumption and active energy metabolism [41,42]. HEK293 cells were cultured in CCM in comprising Dulbecco’s modified essential medium (DMEM) supplemented with 2 mM l-glutamine, 1% pen-strep-fungizone (500 units potassium penicillin, 500 μg streptomycin, 1.25 μg amphotericin B/5 mL flask), 10 mM HEPES and 10% fetal bovine serum in 25 cm^3^ flasks at 37 °C; 5% CO_2_. HEK293 cells are established from primary human embryonic kidney and have the capacity to switch metabolic processes from glucose consumption and lactate production to glucose–lactate co-consumption [43]. This characteristic makes HEK293 cells an ideal model to study potential changes to glucose metabolism as a result of PAT exposure.

### 5.2. Dosage Information

PAT (5 mg) was dissolved in 1ml 100% dimethyl sulfoxide (DMSO) (32 mM PAT). Smaller PAT stock solutions were prepared in 0.1 M PBS to a final concentration of 1 mM. When 90% confluent, cells were serum starved for 16 h (h) and then treated with PAT (0.2 μM; 2.0 μM) in 5 mL CCM for 24 h. These PAT concentrations are below and above the safety level of PAT in consumables, respectively, and were determined from incidence and monitor studies [3,5]. These studies indicate that despite a safety level of 50 µg/L (0.3 µM), PAT exposure and levels in consumables can vary from 0.1 µM to 4.0 µM. Cells grown and treated under these conditions (normoglycemic: 5 mM glucose) are referred to as NG in the results.

To determine the effect of PAT on glucose tolerance, once 90% confluent HepG2 and HEK293 cells were serum starved for 16 h and exposed to high glucose CCM (HG) (25 mM) and PAT (0.2 μM; 2.0 μM) for 24 h. 

Finally, to determine PAT effect on insulin signaling, 90% confluent HepG2 cells were serum starved for 16 h, exposed to NG (5 mM glucose) or HG (25 mM glucose) media and PAT (0.2 μM; 2.0 μM) for 24 h. Cells were then stimulated with 10 ng/mL (1.7 nM) insulin for 5 min before the media was removed. These are represented by NG i+ and HG i+ in the results. HEK293 cells show a limited insulin signaling profile and were excluded from this parameter in the study. 

Metformin (5 mM) was used as a positive control in all protein expression experiments; this concentration was selected from the literature [44]. 

Results are representative of three independent experiments completed in triplicate.

### 5.3. Quantitative Polymerase Chain Reaction (PCR)

#### 5.3.1. RNA Isolation and cDNA Preparation

RNA was isolated adding 500 μL QIAzol lysis reagent (Qiagen, Hilden, Germany) to 1 × 10^6^ cells in 500 μL 0.1 M PBS and incubated overnight (−80 °C). Chloroform (100 µL) was then added and samples were centrifuged (15 min, 12,000× *g*, 4 °C). Isopropanol (250 µL) was added to the aqueous phase in a clean tube and incubated overnight (−80 °C). Samples were centrifuged (20 min, 12,000× *g*, 4 °C), supernatant was discarded and pellets were washed with 500 µL cold ethanol and centrifuged (15 min, 7400× *g*, 4 °C) once more. Ethanol was then aspirated; samples were dried and resuspended in 12.5 µL RNase-free water. RNA was quantified using the Nanodrop2000 and standardized to 1000 µg/μL. cDNA was synthesized using Bio-Rad iScript™ reaction mix as per the manufacturer’s instructions (iScript™ cDNA Synthesis kit Bio-Rad; 107-8890, Johannesburg, South Africa). Thermocycler conditions were 25 °C for 5 min, 42 °C for 30 min, 85 °C for 5 min and a final hold at 4 °C. 

#### 5.3.2. mRNA Quantification

mRNA levels of glucose homeostasis enzymes *PYGL, PFK-1* and *PCK-1* were evaluated using the SsoAdvanced™ Universal SYBR Green Supermix (Bio-Rad; 170-880) according to the manufacturer’s instructions. Once synthesized (as above), a reaction volume made up to 25 µL comprising cDNA template, 30 nM sense primer, 30 nM antisense primer, reaction mix and nuclease-free water was made up. Primer sequences and annealing temperatures were as follows *PYGL* [Sense 5′-TGCCCGGCTACATGAATAACA-3′; Antisense 5′-TGTCATTGGGATAGAGACCC-3′; 56.5 °C]; *PCK-1* [Sense 5′-AAAACGGCCTGAACCTCTCG-3′; Antisense 5′-ACACAGCTCAGCGTTATTCTC-3′; 56.5 °C]; *PFK-2* [Sense 5′AGTCCTACGACTTCTTTCGGC-3′; Antisense 5′-TCTCCTCAGTGAGATACGCCT-3′; 57 °C] qPCR was completed using CFX Touch^™^ Real Time PCR Detection System (Bio-Rad, Johannesburg, South Africa). The reaction was subjected to an initial denaturation (95 °C, 4 min), followed by 37 denaturation cycles (95 °C, 15 s), annealing (primer-specific temperature, 40 s), extension (72 °C, 30 s) and a plate read for 37 cycles. *β actin* [Sense 5′-TGACGGGTCACCCACACTGTGCCCAT-3′; Antisense 5′-CTAGAAGCATTTGCGGTGGACGATGGAGGG-3′] was run under the same conditions and used as the housekeeping gene. Data were analyzed using the method described by Livak and Schmittgen and represented as fold change (2^−ΔΔCT^) relative to the control [45].

### 5.4. Western Blotting

#### 5.4.1. Protein Isolation

Cytobuster (Novagen^®^, Pretoria, South Africa) supplemented with protease inhibitor and phosphatase inhibitor (Roche 05892791001 and 04906837001, respectively) was added to cells (4 °C, 10 min) and centrifuged (5 min, 10,000× *g*, 4 °C). Crude protein extracts were quantified using the bichinchonic acid (BCA) assay (Sigma, St. Louis, MO, USA) and standardized to 1 mg/mL. Samples were denatured by boiling (5 min, 100 °C) in Laemmli buffer (dH_2_O, 0.5 M TrisHCl (pH 6.8), glycerol, 10% sodium dodecyl sulphate polyacrylamide (SDS), β-mercaptoethanol, 1% bromophenol blue). 

#### 5.4.2. Sodium Dodecyl Sulphate–Polyacrylamide Gel Electrophoresis (SDS-PAGE) and Immunoblotting

Samples were separated by electrophoresis on 7% SDS gels (1 h, 150 V) and transferred to nitrocellulose membranes using the TransBlot Turbo System^®^ (Bio-Rad, Johannesburg, South Africa). Membranes were blocked (1 h, 3%, bovine serum albumin (BSA)) in Tris-buffered saline with Tween20 (TTBS) (20 mM Tris–HCl; pH 7.4), 500 mM NaCl and 0.01% Tween 20). Membranes were then incubated with primary antibody (phosphor-tyrosine (p-tyr100) (CST#9411); phosphor-GSK3β (ser 9) (pGSK3) (ab107166); total GSK3α/β (ab15314); GLUT2 (ab54460); p44/42 MAPK (ERK1/2) (CST#9102); phosphor-p44/42 MAPK (ERK1/2) (Thr202/Try204) (CST#9106); PI3K (CST#5405); phosphor-ser473 Akt (CST#9271); Akt (CST#9272); PDK-1 (ab110025); phosphor-PDH (E1α) (ab92696); PDH (E1α) (ab110330); (1:1000)) in 1% BSA in TTBS overnight at 4 °C. Membranes were washed four times (10 mL, TTBS, 10 min) and treated with horseradish peroxidase-conjugated secondary antibody (anti-rabbit, CST #7074; anti-mouse, CST #7076, 1:10,000) in 1% BSA for one hour at room temperature. Membranes were then washed four times (10 mL, TTBS, 10 min) and immunoreactivity was detected (Clarity™ Western ECL Blotting Substrate, Bio-Rad, Johannesburg, South Africa) with the Bio-Rad ChemiDoc Imaging System. Protein bands were analyzed with the Bio-Rad Image Lab Analysis 6.0 software and normalized against the corresponding β-actin bands.

### 5.5. Statistical Analysis

Results are represented as mean fold change ± standard deviation (SD) relative to normalized control. Statistical significance was assessed using one-way ANOVA with appropriate post hoc comparisons on GraphPad Version 5.0 Software. *p* values less than 0.05 were considered significant. 

## Figures and Tables

**Table 1:** Extracellular lactate levels (mean ± SD) measured following 24 h PAT exposure in HepG2 and HEK293 cells.

Treatment	Lactate Levels (mmol/L) Normoglycemic Media (NG)	Lactate Levels (mmol/L) Hyperglycemic Media (HG)
HEK293 Control	8.85 ± 0.1306	17.78 ± 0.07348
HEK293 0.2 μM PAT	7.77 ± 0.1796	12.92 ± 0.1878
HEK293 2.0 μM PAT	7.75 ± 0.3103	15.97 ± 0.1470
HepG2 Control	3.36 ± 0.2531	5.58 ± 0.2368
HepG2 0.2 µM PAT	2.64 ± 0.1551	5.35 ± 0.3101
HepG2 2.0 µM PAT	3.12 ± 0.1796	5.17 ± 0.8410
HepG2 (i+) Control	3.15 ± 0.1388	3.63 ± 0.07348
HepG2 (i+) 0.2 µM PAT	2.79 ± 0.1470	3.69 ± 0.4736
HepG2 (i+) 2.0 µM PAT	2.40 ± 0.04899	3.68 ± 0.2286

## Data Availability

Not applicable.
